# Determining the Quality Factor of Dielectric Ceramic Mixtures with Dielectric Constants in the Microwave Frequency Range

**DOI:** 10.1038/s41598-017-14333-9

**Published:** 2017-10-26

**Authors:** Hetuo Chen, Xuewen Fu, Qi An, Bin Tang, Shuren Zhang, Hao Yang, Yin Long, Mark Harfouche, Huolei Wang, Yingxiang Li

**Affiliations:** 1 State Key Laboratory of Electronic Thin Films and Integrated Devices, University of Electronic Science and Technology of China, Chengdu, Sichuan China; 2 Division of Engineering and Applied Science, California Institute of Technology, Pasadena, California, USA; 3 Department of Chemical and Materials Engineering, University of Nevada, Reno, Nevada USA; 4 Division of Chemistry and Chemical Engineering, California Institute of Technology, Pasadena, California, USA

## Abstract

Microwave dielectric ceramic materials are extensively utilized in microwave applications because of their high dielectric constants and quality factors. These applications also require ceramics of zero temperature coefficients at the resonant frequency (*τ*_*f*_), which can be realized through mixing a ceramic that one is interested in with another ceramic with −*τ*_*f*_, or by performing the ionic substitution. With the mixing/ionic substitution, it is indispensable to compute the quality factors precisely. Previous study indicates that the quality factor depends on the grain size, porosity, internal strain, structure, phase evolution, and conductivity *etc*. Here we derive a quality factor formula based on the definition, which works very well for multiphase composites, single phase solid solutions, and equivalent ionic substituted single phase materials. Our formula calculation and fits to the previous experimental results demonstrate that the quality factor of the ceramic mixtures strongly depend on the dielectric constants and the dielectric constant variation index. Our results suggest that the impacts from grain size, porosity, and internal strain *etc*. can be summarized to the dielectric constant or dielectric constant variation index, which is of great importance for future design of high performance microwave dielectric ceramics.

## Introduction

Microwave dielectric ceramics have been used extensively in resonators, filters, radars, and integrated passive modules for wireless communication applications^1–3^. These applications demand ceramics with high dielectric constant (*ε*_*r*_), high quality factor (*Q*) and near-zero temperature coefficient at the resonant frequency (*τ*_*f*_)^1–3^. However, most ceramics inherently possess non-zero temperature coefficients at the resonant frequency^3,4^. Therefore, for a ceramic of interest in real applications, another ceramic with *τ*_*f*_ of an opposite sign needs to be mixed with it or ionic substitution in it is required, to modify the *τ*_*f*_ to zero^5,6^. After the mixing or inion substitution, it is a big challenge to precisely calculate the quality factor^5–9^.

The dielectric constant and the quality factor are usually calculated separately^1,5–9^. When the material of −*τ*_*f*_ is mixed, a binary phase composite could be formed if the structure of the two ceramics are different^6,8–16^. Otherwise, the mixed material will be a single phase solid solution^4,5,17–24^. In previous work, the solid solution was treated as a two-phase composite^16,17,20–24^, where the dielectric constant can be derived from the Maxwell-Wagner equation^7–9,25,26^

1 $${{\varepsilon }_{r}}^{k}=(1-V){\varepsilon }_{r1}^{k}+V{\varepsilon }_{r2}^{k}$$

where *V* represents the volume molar ratio of the second material, *ε*_*r*_ is the dielectric constant of the composite, *ε*_*r*1_ and *ε*_*r*2_ are the dielectric constants of the two ceramics, respectively. By using a parallel or serial capacitor model, the parameter *k* in equation (1) corresponds to +1 and −1, individually^7^. Similarly, other different models have also been proposed such as Lichtenecker logarithmic model^7,9^, Jayasundere-Smith formula^8^, and Maxwell spherical particle model^27^. However, these models were derived from the basis of different assumptions. For example, the Maxwell spherical particle model assumed that the second material consists of spherical particles and these particles distribute randomly in the matrix. When the doping concentration is very high, to eliminate the deviation between the experimental results and the Maxwell spherical particle model or the Lichtenecker logarithmic model, the Jayasundere-Smith model considers the impact from neighbor spheres^9^. Thus, the experimental dielectric constants may deviate largely from calculated results from these models if these assumptions are not satisfied, e.g. the Jayasundere-Smith model does not work well when the doping concentration is low^7–9^.

**Figure 1:**
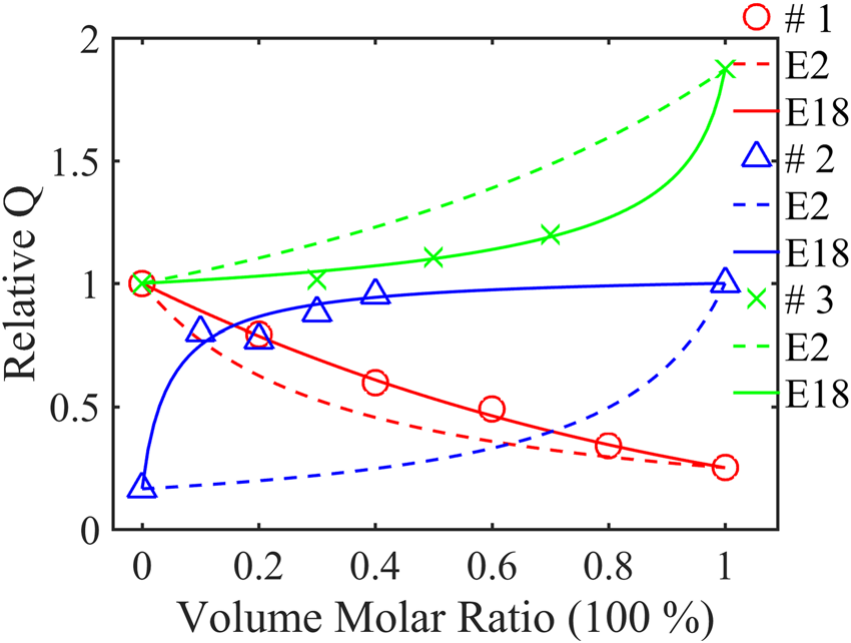
Reported relative quality factors (individual samples) and calculated relative quality factors via Equation (2) (dashed lines, E2) and Equation (18) (solid lines, E18): # 1, red, BaNd_2_Ti_4_O_12_-BaZn_2_Ti_4_O_11_ multiphase composition; # 2, blue, CaTiO_3_-NdAlO_3_ perovskite structure solid solution; # 3, green × and lines (Mg_0.95_Ni_0.05_)_4_(Nb_1−x_Ta_x_)_2_O_9_ equivalent ionic substitution.

The quality factor of the binary phase composite can be estimated by^4,8,28^

2 $${Q}^{-1}=(1-V){Q}_{1}^{-1}+V{Q}_{2}^{-1}$$

where *Q* represents the quality factor of the mixture system, and *Q*_1_ and *Q*_2_ are the quality factors of the two ceramics, respectively. From the definition of *Q* (it will be given in equation (8) in the next section) at the microwave frequency range, the quality factor of the mixture is related to both the dielectric constant and the resonant frequency^1,5,6,25,26,29^. Equation (2) only considers the quality factor of the two initial materials and is a simple superposition relationship. Figure 1 shows the comparison of the reported experimental quality factors with the calculated values via equation (2)^23,30,31^. The trend of calculated results (dashed lines) is roughly consistent with the reported experimental data (separated markers) of BaNd_2_Ti_4_O_12_-BaZn_2_Ti_4_O_11_ binary phase composite and (Mg_0.95_Ni_0.05_)_4_(Nb_1-x_Ta_x_)_2_O_9_ ionic substitution. However, the reported experimental data cannot be predicted by equation (2) for CaTiO_3_-NdAlO_3_ solid solution. Apparently, equation (2) is not a general model that cannot predict well the quality factor of many other binary ceramic systems^6,7,23,30,31^.

The solid solution can be treated as the ionic substitution situation at some circumstances^4,18,19^. When the ionic substitution is performed, the original phase can be maintained. Shannon *et al*. has reported that the dielectric constant can be calculated through the Clausius-Mosotti equation, but the equation requires the molecule or ion with a cubic symmetry^2,32–35^. As a result, only the tendency of dielectric constant variation can be roughly estimated if the molecule or ion with non-cubic symmetry^2,5,35^. The quality factor change of the ionic substitution is usually attributed to the grain size^20,21,23^, porosity^11,12^, structure^24^, phase evolution^5^, internal strain^2,36–38^, material conductivity^39^ and so on. Huang^10^ *et al*. reported that larger grain size corresponds to less grain boundaries per unit volume, which leads to higher quality factor because of periodicity symmetry breaking and less two-dimensional defects. Zhou^5,6^ *et al*. reported the impact of structure and phase evolution on the quality factor in BiVO_4_ based ceramics. Penn and Alford^27^ *et al*. computed the relation between the porosity and quality factor by fitting the measured data. Ohsato^37^ linked the quality factor to the internal strain based on the work of Stokes^38^ *et al*. Kuang^39^ evaluated the quality factor by estimating the materials’ conductivity at microwave frequency. In all these reports, quality factor trends agree with the grain size, phase evolution, porosity, internal strain, and conductivity^5,6,10,27,37–39^. However, precise calculation of the quality factor of mixed ceramic system is still illusive. Especially when the extra new phase(s) appears, it would complex the calculation of the quality factor^40^.

The quality factor of the mixed (including binary phase, solid solution and equivalent ionic substitution) ceramics should be predicable if we know the microwave properties (including dielectric constants, quality factors, and the volume molar ratios) of the two materials defined in equation (1) and (2), and the new phase(s). Because the volume molar ratio of the extra new phases was not reported in previous literatures^40–43^, the examples presented in this paper do not include the case where extra phases exist. We first revise the Maxwell-Wagner equation and then derive a precise *Q* formula by the definition. Then we apply the formula to fit the dielectric constant and calculate *Q* over 30 groups of examples and compare them with the reported experimental results. Our theoretical results agree very well with the reported experimental measurements, validating our new formula and assumption. We find that the precise quality factor calculation strongly depends on the dielectric constant when the materials in each group are fabricated by the same or similar process. In addition, the situations, where new extra phase appears and material’s conductivity dominates the dielectric loss, will also be discussed.

## Theory

The complex dielectric constant of a ceramic is given by the classical harmonic oscillator model^1,5,6,25,26,29^

3 $$\varepsilon (\omega )={\varepsilon }_{\infty }+\sum _{j=1}^{n}\frac{{\omega }_{pj}^{2}}{{\omega }_{oj}^{2}-{\omega }^{2}-i{\gamma }_{j}\omega }$$

where *ε*_∞_ is a constant permittivity of the material, *n* represents the number of Lorentz oscillators, *ω* is the working frequency at GHz, *i* ^2^ = −1; while *ω*_*pj*_, *ω*_*oj*_, and *γ*_*j*_ are the plasma frequency, natural resonant frequency, and damping factor of the *j*-th Lorentz oscillator, respectively. If one separates the real (*ε*_*r*_) and imaginary (*ε*_*i*_) parts of equation (3) as^25,26,29^

4 $$\varepsilon (\omega )={\varepsilon }_{r}+i{\varepsilon }_{i}$$

then the real and imaginary parts of the complex dielectric constant are given by^29^

5 $${\varepsilon }_{r}={\varepsilon }_{\infty }+\sum _{j}^{n}{\omega }_{pj}^{2}\frac{{\omega }_{oj}^{2}-{\omega }^{2}}{{({\omega }_{oj}^{2}-{\omega }^{2})}^{2}+{\gamma }_{j}^{2}{\omega }^{2}}$$

6 $${\varepsilon }_{i}=\sum _{j}^{n}{\omega }_{pj}^{2}\frac{{\gamma }_{j}\omega }{{({\omega }_{oj}^{2}-{\omega }^{2})}^{2}+{\gamma }_{j}^{2}{\omega }^{2}}$$

The dielectric loss (tan*δ*) of a ceramic is defined as^1,5,6,29^

7 $$\tan \,{\delta }=\frac{{\varepsilon }_{i}}{{\varepsilon }_{r}}$$

Since *ω*_*oj*_/*ω* is ~10^3^, equation (7) can be rewritten as^1,5,6,25,26,29^

8 $$\tan \,{\delta }\approx \omega \frac{\sum _{j}^{n}\frac{{\gamma }_{j}}{{\omega }_{oj}^{2}}}{{\varepsilon }_{\infty }+\sum _{j}^{n}\frac{{\omega }_{pj}^{2}}{{\omega }_{oj}^{2}}}$$

Based on equation (8), the dielectric loss of a ceramic is frequency dependent^1^. Therefore, the imaginary part of the dielectric constant for single phase ceramics can be determined by the real part of the dielectric constant if the measurement frequency is given.

Considering the ceramic mixture as a multiple phase system, the dielectric loss is determined by the ratio between the imaginary and real parts of the dielectric constant of the mixture based on equation (7). In this case, the real part of the dielectric constant can be predicted by the revised Maxwell-Wagner equation^7,8,25,26^

9 $${{\varepsilon }_{r}}^{k}=\sum _{l=1}^{m}{V}_{l}{\varepsilon }_{rl}^{k}$$

where *l* is the *l*-th ceramic, *V*_*l*_ is its volume molar ratio, *ε*_*rl*_ is its dielectric constant, and *m* means the number of ceramics mixed. A *k* value can be obtained through fitting the experimental data with equation (9). If the frequency *ω* is known, the ratio between the imaginary and real part of the dielectric constant is a constant according to equation (7). Without loss of generality, we assume that the imaginary dielectric constant of the mixture obeys the same rule as equation (9) and can be written as

10 $${{\varepsilon }_{i}}^{k}=\sum _{l=1}^{m}{V}_{l}{\varepsilon }_{il}^{k}$$

where *l* is the *l*-th ceramic, *V*_*l*_ is its volume molar ratio, *ε*_*il*_ is its imaginary dielectric constant, and *m* means the number of ceramics mixed. Therefore, the dielectric loss of the mixture can be obtained by substituting equations (9) and (10) into equation (7)

11 $${(\tan {\delta })}^{k}=\frac{\sum _{l=1}^{m}{V}_{l}{\varepsilon }_{il}^{k}}{\sum _{l=1}^{m}{V}_{l}{\varepsilon }_{rl}^{k}}$$

Using equation (7) again for each *l*, equation (11) can be recast as

12 $${(\tan {\delta })}^{k}=\frac{\sum _{l=1}^{m}{V}_{l}{(\tan {{\delta }}_{l})}^{k}{\varepsilon }_{rl}^{k}}{\sum _{l=1}^{m}{V}_{l}{\varepsilon }_{rl}^{k}}$$

where *l* is the *l*-th ceramic, tan*δ*_*l*_ is its dielectric loss and tan*δ* is the dielectric loss of the mixture. In practice, quality factor is approximately the reciprocal of the dielectric loss^1,5,6,25,29^

13 $${Q}^{-1}=\,\tan \,{\delta }$$

Substituting equation (13) into (12), one obtains

14 $${Q}^{-k}=\frac{\sum _{l=1}^{m}{V}_{l}{Q}_{l}^{-k}{\varepsilon }_{rl}^{k}}{\sum _{l=1}^{m}{V}_{l}{\varepsilon }_{rl}^{k}}$$

where *Q* is the quality factor of the mixing phase, *Q*_*l*_ is the quality factor of the *l*-th ceramic. According to equation (14), the quality factor of a microwave ceramic mixture depends on the quality factor and dielectric constants of the initial ceramics and the fitting parameter *k* (in this study we name it as the dielectric constant variation index). Figure 1 shows the computed *Q* values via equation (14) and (18), which match very well with the reported experimental data for the binary phase composite, solid solution, and equivalent ionic substitution examples.

Generally speaking, the target of mixing/ionic substitution is to adjust the properties of the first material (*l* = 1 in equations (9) and (14)). By defining relative dielectric constant ${R}_{{\varepsilon }_{r}}=\frac{{\varepsilon }_{r}}{{\varepsilon }_{r1}}$, dielectric constant ratio ${R}_{{\varepsilon }_{rl1}}=\frac{{\varepsilon }_{rl}}{{\varepsilon }_{r1}}$, relative quality factor ${R}_{Q}={\frac{Q}{Q}}_{1}$, and quality factor ratio ${R}_{{Q}_{1l}}=\frac{{Q}_{1}}{{Q}_{l}}$, equations (9) and (14) can be rewritten as

15 $${R}_{{\varepsilon }_{r}}^{k}=\sum _{l=1}^{m}{V}_{l}{R}_{{\varepsilon }_{rl1}}^{k}$$

and

16 $${R}_{Q}^{-k}=\frac{\sum _{l=1}^{m}{V}_{l}{R}_{{Q}_{1l}}^{k}{R}_{{\varepsilon }_{rl1}}^{k}}{\sum _{l=1}^{m}{V}_{l}{R}_{{\varepsilon }_{rl1}}^{k}}$$

Through equation (16), both the dielectric constant and the quality factor of the mixture are compared to the first material (*l* = 1 in equations (9) and (14)). With this manipulation, we can conveniently compare the dielectric constants’ variation of different material groups. In addition, quality factors of the situations, where new extra phase appears can also be precisely determined by equation (16) when the volume molar ratio of the new phase, can be determined.

In the past decade, the experimental *Q* of two ceramics mixing^6,9–16^ and one ion substitution^5,44–50^ have been extensively reported. These data of the reported binary systems can be verified with our theory, where the parameter *m* is 2 in equations (15) and (16). In the following, examples will be limited to the situation of two ceramics mixing or one ionic substitution. In this situation, *R*_*Q*11_ = 1, *Rε*_*r*11_ = 1 and equations (15) and (16) become

17 $${R}_{{\varepsilon }_{r}}^{k}=1-V+V{R}_{{\varepsilon }_{r21}}^{k}$$

and

18 $${R}_{Q}^{-k}=\frac{1-V+V{R}_{{Q}_{12}}^{k}{R}_{{\varepsilon }_{r21}}^{k}}{1-V+V{R}_{{\varepsilon }_{r21}}^{k}}$$

The *k* value of each group will be obtained through fitting the measured dielectric constant with equation (17). The quality factor will be computed via equation (18) and will be compared to the reported experimental results in the literatures. Examples will be grouped into: (1) binary phase composites, where the dielectric constant and quality factor of the two original materials are known; (2) single phase solid solution, e.g. the quality factor of the perovskite structure CaTiO_3_-NdAlO_3_ will be calculated through dielectric constants and quality factors of CaTiO_3_ and NdAlO_3_^32^; (3) equivalent ionic substitutions, e.g. the quality factors of Ca_1-x_Sr_x_TiO_3_ (0 ≤ *x* ≤ 1) will be calculated via CaTiO_3_ and SrTiO_3_^49^. According to equation (17), R*ε*_*rl*_ can be any number. In the examples of this paper, the value of R*ε*_*rl*_ (0 < R*ε*_*rl*_ < 1) will be revised to be R*ε*_*rl*_ > 1 by exchanging their orders for clear comparison. The examples will be separated by R*ε*_*rl*_, near 1 or much larger than 1. We will discuss but will not exemplify the in-equivalent ionic substitution where the quality factor is limited by material conductivity^39,51^. In addition, the case of extra new phases co-existing will not be considered because the volume molar ratios were not given in the previous literatures^41–43^.

## Results and Discussions

### Theoretical calculation

**Figure 2:**
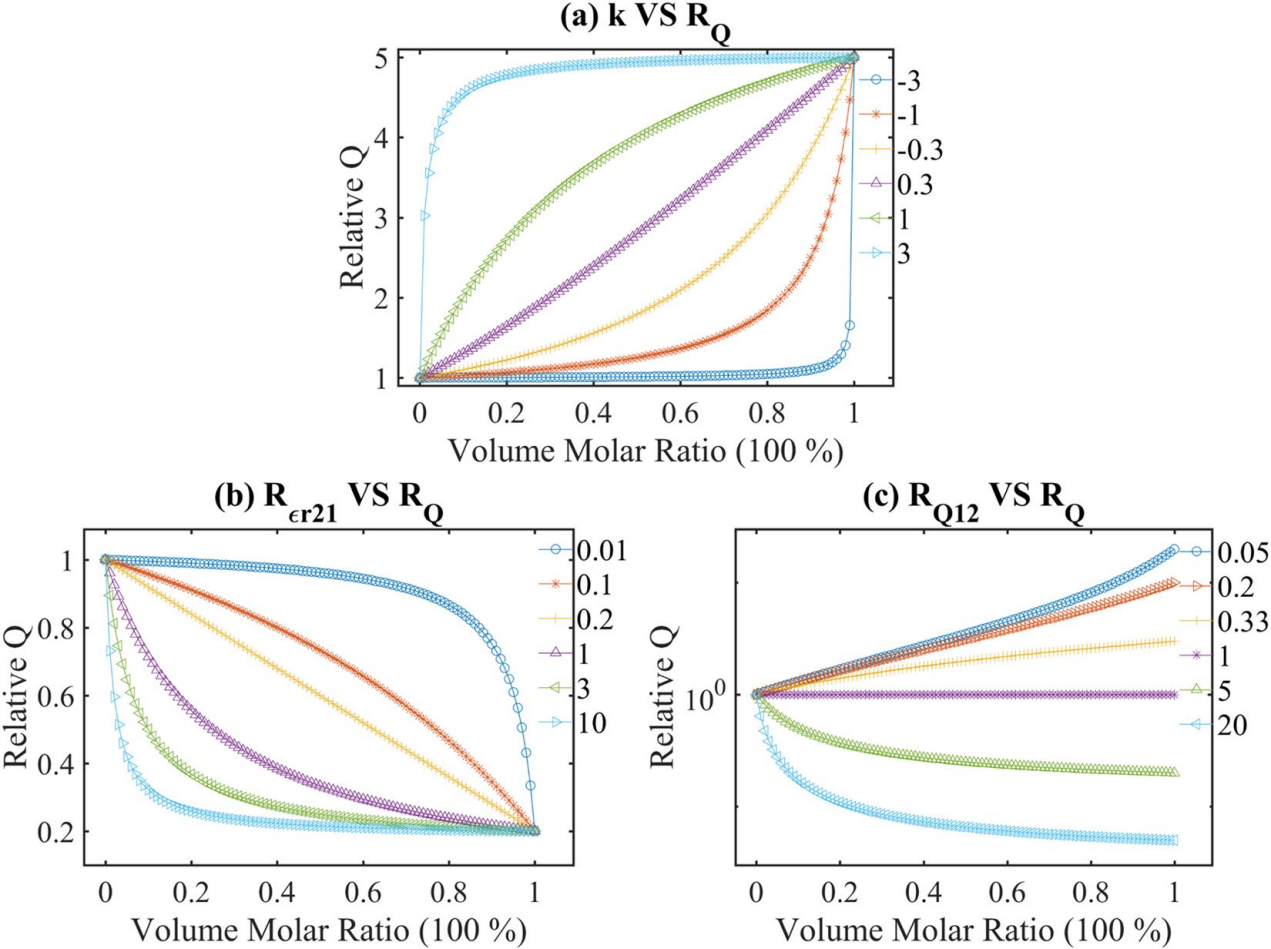
Theoretical calculation of Equation (18): (**a**) *Rε*_*r*21_ = 3, *R*_*Q*12_ = 5, the dependence of relative quality factor *R*_*Q*_ on *k* values, versus the volume molar ratio *V*; (**b**) *k* = 1, *R*_*Q12*_ = 5, the dependence of relative quality factor *R*_*Q*_ on *Rε*_*r*21_, versus the volume molar ratio *V*; (c) *k* = 1, *Rε*_*r*21_ = 3, the dependence of relative quality factor *R*_*Q*_ on *R*_*Q*12_, versus the volume molar ratio *V*.

According to equation (18), the relative quality factor is determined by four variants: *Rε*_*r2*1_, *R*_*Q*1*2*_, *V*, and *k*. To show the relation between the relative quality factor and each variant as a function of volume molar ratios, we randomly fix two of these variants. Figure 2(a) shows the dependence of relative quality factor *R*_*Q*_ on *k* values as a function of the volume molar ratio *V* if *Rε*_*r*21_ = 3 and *R*_*Q*12_ = 5. In general, when *k* is smaller than 0.3, *R*_*Q*_ shows a concave increase trend. If *k* is negative and very small, e.g. *k* = −3, *R*_*Q*_ will increase slowly first until *V* is around 0.9 and then there is a sharp increase with the increase of the volume molar ratio. Similarly, if *k* is positive and very large, e.g. *k* = 3, *R*_*Q*_ exhibits a sharp increase around *V* = 0.1, then it slowly increases to 5. As *k* gets around 0.3, *R*_*Q*_ will increase gradually to 5 and the sharp trend disappears with the increase of the volume molar ratio. Variation trend of *R*_*Q*_ will change accordingly as *Rε*_*r2*1_ and *R*_*Q*1*2*_ are fixed as other numbers.

Figure 2(b) displays the dependence of relative quality factor on the dielectric constant ratio *Rε*_*r*21_, with the volume molar ratio *V* increasing from 0 to 1 when *k* = 1 and *R*_*Q12*_ = 5. In general, when *Rε*_*r*21_ is smaller than 0.2, *R*_*Q*_ shows a convex decrease, or it drops concavely. If *Rε*_*r*21_ is very small, e.g. *Rε*_*r*21_ = 0.01, *R*_*Q*_ decreases slowly first and then shows a sharp decrease for *V* of about 0.8 as the volume molar ratio increases. Similarly, if *Rε*_*r*21_ is very large, e.g. *Rε*_*r*21_ = 10, *R*_*Q*_ exhibits a sharp decrease around *V* = 0.1, then it slowly decreases to 0.2. As *Rε*_*r*21_ gets around 0.2, *R*_*Q*_ will decrease gradually to 0.2 and the sharp trend disappears as the volume molar ratio increases. Variation trend of *R*_*Q*_ will change accordingly as *Rε*_*r2*1_ and *k* are fixed as other numbers.

Figure 2(c) shows the dependence of relative quality factor on the quality factor ratio *R*_*Q*12_, as the volume molar ratio *V* increases from 0 to 1 for *k* = 1 and *Rε*_*r*21_ = 3. In order to show the situations of *R*_*Q*12_ < 1, the y axis is set in log-scale. When *R*_*Q*12_ > 1, *R*_*Q*_ decreases versus *V* while when *R*_*Q12*_ < 1 it increases. *R*_*Q*_ will maintain a constant if *R*_*Q*12_ = 1.

In summary, the relative quality factor is determined by *V*, *R*_*Q*12_, *k* and *Rε*_*r*21_. These parameters are set as above to show the systematical variation, which can be also set as other numbers if necessary. However, their relation is confined by equation (17). *k* and *Rε*_*r*21_ determine a concave or convex trend of the relative quality factor versus *V*, while *R*_*Q*12_ determines whether the relative quality factor increase or decrease.

In most previous literatures, data of *Q* × *f* were reported, not *Q* ^1,2,5,6,9–24^. In ref.^52^, the resonant frequency is reversely proportional to the size of a cylinder and its dielectric constant. The resonant frequency (*f*) in these examples can be fixed to a number such as 4, and it is given by^52^

19 $$f\propto \frac{1}{\sqrt{{\varepsilon }_{r}}}$$

The samples’ sizes are assumed un-changed as the volume molar ratio varies in all the reports. Then *Q* can be extracted from the *Q* × *f* data.

In the following 3 groups of examples, *k* values will be obtained by fitting the reported dielectric constants with equation (17). The relative quality factors of each group will be computed through equation (18) by substituting *k* into it. The computed quality factor will be compared to the reported experimental results. Typical microwave ceramics are included, e.g. MgTiO_3_^10,53,54^, BaTi_4_O_9_^55^, Ba_6-3x_Nd_8+2x_Ti_18_O_54_^4,30,48^, TiO_2_^6,8,9,13,15,56,57^, CaTiO_3_^11,14,16,22,24,49,50,58,59^, and Li_1/2_Nd_1/2_TiO_3_^17,20,22^. These 3 groups are: 1. multiphase composites, 2. single phase solid solutions, 3. equivalent ionic substituted single phase ceramics. Only the highest *Q* × *f* values will be used because the impact of sintering temperature can be minimized.

### Multiphase examples

**Figure 3:**
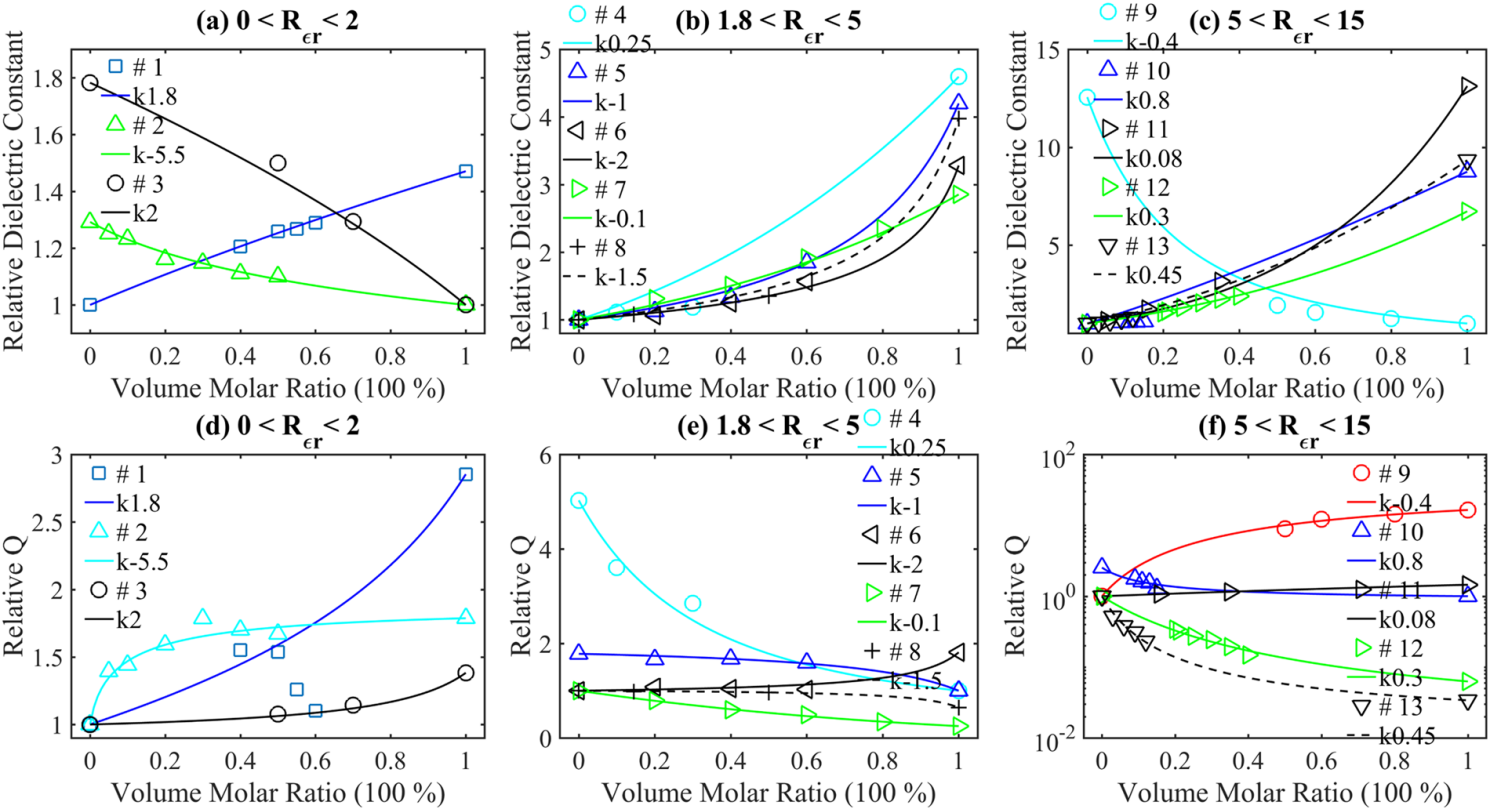
Multiphase examples: (**a**–**c**) the comparison between the reported relative dielectric constants (individual samples) and calculation from Equation (17) (solid lines); (**d**–**f**) the comparison between the reported relative quality factors (individual samples) and calculation from Equation (18) (solid lines). (**a**) and (**d**) # 1. blue squares and lines, *k* = 1.8, BiVO_4_-TiO_2_; # 2. green triangles and lines, *k* = −5.5, BaTi_4_O_9_-BaZn_2_Ti_4_O_11_; # 3. black circles and lines, *k* = 2, MgTiO_3_-MgTa_2_O_6_; (**b**) and (**e**) # 4. blue-green circles and lines, *k* = 0.25, Ca_4_MgNb_2_TiO_12_-CaTiO_3_; # 5. blue triangles and lines, *k* = −1, ZnNb_2_O_6_-TiO_2_; # 6. black triangles and lines, *k* = −2, ZnTa_2_O_6_-TiO_2_; # 7. green triangles and lines, *k* = −0.1, BaNd_2_Ti_4_O_12_-BaZn_2_Ti_4_O_11_; # 8. black + and dashed lines, *k* = −1.5, Li_2_ZnTi_3_O_8_-TiO_2_; (**c**) and (**f**) # 9. blue-green circles and line in (**c**) and red circles and line in (**f**), *k* = −0.4, Mg_4_Nb_2_O_9_-CaTiO_3_; # 10. blue triangles and lines, *k* = 0.8, BaMg_2_V_2_O_8_-TiO_2_; # 11. black triangles and lines, *k* = 0.08, ZnMoO_4_-TiO_2_; # 12. green triangles and lines, *k* = 0.3, Mg_0.95_Zn_0.05_TiO_3_-Ca_0.6_La_0.8/3_TiO_3_; # 13. black triangles downward and dashed lines, *k* = 0.45, Mg_2_Ti_0.95_Sn_0.05_O_4_-CaTiO_3_.

It is well known that the quality factors of the multiphase ceramics are mainly determined by the two ceramics when they are sintered at the temperature, where the dielectric constants reach peak values versus the volume molar ratio^6,10–16,30,54–56^. However, many reports also accused the variation of *Q* × *f* to dielectric constant^6^, abnormal grain growth^10^, density^11,14,16^, lattice defect concentration^13^, microtopography^30^, phase constitution^56^, packing fraction^15^, reacting element evaporation^54^, and so on. According to equations (8) and (19), *Q* × *f* is independent of the dielectric constant but the quality factor *Q* depends on it. As the volume molar ratio between two ceramics varies, it is reasonable to observe some change in the grain size, density, and theoretical packing fraction *etc*. However, how these changes impact *Q* × *f* values has not been proved. The reacting element evaporation can be treated as the situation of extra new phases appear. In this part, using the dielectric constants and the quality factors of the two starting ceramics, we will show that the quality factor versus the volume molar ratio can be precisely computed via equation (18). To show better comparison, the 13 examples in the first group are separated into 3 figures by the dielectric constant ratio between the two ceramics. The *k* value of each group will be obtained through fitting the reported dielectric constant with equation (9). The examples and their corresponding *k* values are separated in Group I (*Rε*_*r*21_ ~ 1–2): 1. BiVO_4_-TiO_2_, *k* = 1.8^6,56^; 2. BaTi_4_O_9_-BaZn_2_Ti_4_O_11_, *k* = −5.5^55^; 3. MgTiO_3_-MgTa_2_O_6_, *k* = 2^10^; Group II (*Rε*_*r*21_ ~ 2–5): 4. Ca_4_MgNb_2_TiO_12_-CaTiO_3_, *k* = 0.25;^11^ 5. ZnNb_2_O_6_-TiO_2_ *k* = −1^12^; 6. ZnTa_2_O_6_-TiO_2_ *k* = −2^13^; 7. BaNd_2_Ti_4_O_12_-BaZn_2_Ti_4_O_11_, *k* = −0.1^30^; 8. Li_2_ZnTi_3_O_8_-TiO_2_, *k* = −1.5^57^; Group III (*Rε*_*r*21_ ~ 5–13): 9. Mg_4_Nb_2_O_9_-CaTiO_3_, *k* = −0.4^14^; 10. BaMg_2_V_2_O_8_-TiO_2_, *k* = 0.8^15^; 11. ZnMoO_4_-TiO_2_, *k* = 0.08^6,56^; 12. Mg_0.95_Zn_0.05_TiO_3_-Ca_0.6_La_0.8/3_TiO_3_, *k* = 0.3^54,60^; 13. Mg_2_Ti_0.95_Sn_0.05_O_4_-CaTiO_3_, *k* = 0.45^16^. Examples of *Rε*_*r*21_ smaller than 1 can be transferred to larger than 1 by exchange two materials’ orders. In Fig. 3(a)–(c), it shows the reported dielectric constant data and plot of the calculation via equation (9) of these 13 groups. In Fig. 3(a) and (c), all reported data follow equation (9). In Fig. 3(b), there is one data deviate from the prediction with a relative deviation of around 15%. In general, reported dielectric constants fit well to the results computed via equation (9), which confirms the effectiveness of equation (9). Figure 3(d)–(f) compares the reported relative quality factor with the calculated result with equation (18). As shown in Fig. 3 (e) and (f), the reported data follow the line and distribute evenly along the plot. In Figs 3(d), the reported data points of BiVO_4_-TiO_2_ deviate from the plot, which may be due to their diameter change^6,56^. In ref.^6^, BiVO_4_ can be well sintered at 900 ^o^C while TiO_2_ can be well sintered at around 1500 ^o^C, which indicates that too much TiO_2_ addition would lead to different dimension change. Via equations (8) and (19), this different dimension corresponds to higher resonant frequency and lower quality factor. However, the literature did not supply size information. In general, all reported quality factors follow well with the results computed via equation (18), which indicates that the quality factor of ceramic mixtures is mainly determined by their dielectric constant and dielectric constant variation index *k*. Therefore, using dielectric constants and quality factors of two starting materials, we can precisely compute the quality factor of the multiphase mixtures versus their volume molar ratio. In other words, if the ceramic mixtures are well sintered, the reported factors may actually affect the dielectric constant variation index *k*.

### Single phase solid solution examples

**Figure 4:**
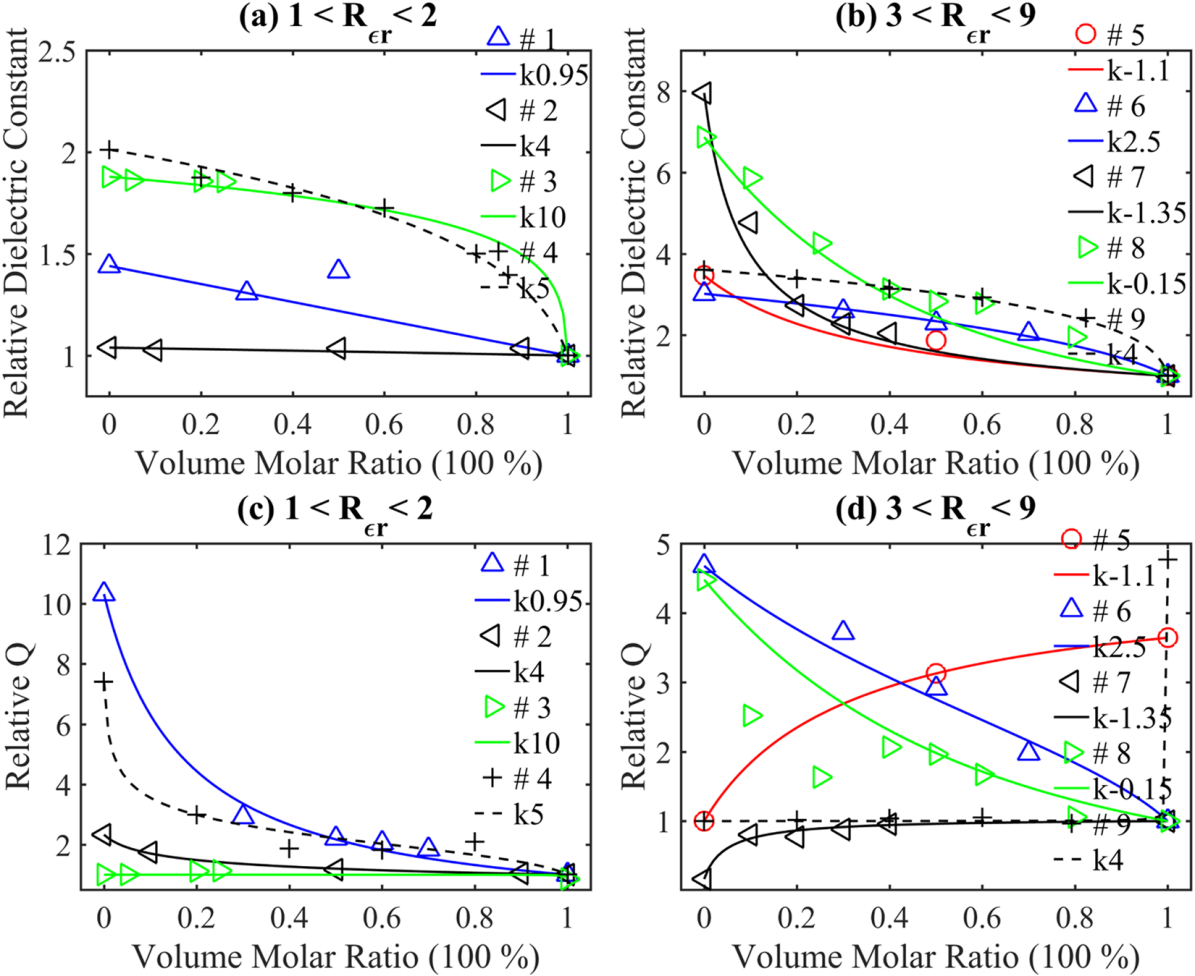
Solid solution examples: (**a**–**b**) the comparison between the reported relative dielectric constants (individual samples) and calculation from Equation (17) (solid lines); (**c**–**d**) the comparison between the reported relative quality factors (individual samples) and calculation from Equation (18) (solid lines). (**a**) and (**c**) # 1. blue triangles and lines, *k* = 0.95, Ca_2/5_Sm_2/5_TiO_3_-Li_1/2_Nd_1/2_TiO_3_; # 2. black triangles and lines, *k* = 4, Ba_2_CaWO_6_-Ba_2_SrWO_6_; # 3. green triangles and lines, *k* = 10, ZnNb_2_O_6_-MgTa_2_O_6_; # 4. black + and lines, *k* = 5, Ca_4/5_Sr_1/5_TiO_3_-Li_1/2_Nd_1/2_TiO_3_; (**b**) and (**d**) # 5. red circles and lines, *k* = −1.1, Ca_3/5_La_0.8/3_TiO_3_-CaMg_1/3_Nb_2/3_O_3_; # 6. blue triangles and lines, *k* = 2.5, CaTiO_3_- Li_1/2_Sm_1/2_TiO_3_; # 7. black triangles and lines, *k* = −1.35, CaTiO_3_-NdAlO_3_; # 8. green triangles and lines, *k* = −0.15, CaTiO_3_-SrMg_1/3_Nb_2/3_O_3_; # 9. black pluses and lines, *k* = 4, BaNd_2_Ti_4_O_12_-NdAlO_3_.

When mixing two materials with a similar structure, the mixture probably forms a single phase solid solution^17–24^. For example, the mixing of perovskite structure CaTiO_3_ and NdAlO_3_ leads to a solid solution with a single perovskite structure^23^. It was reported that the quality factor of solid solutions is determined by lattice anharmonicity^17^, grain size^18,20,21,23^, structure^24^, and phase constitution^4^ etc. Plenty of microstructure characteristics are performed to relate the quality factor variation to one of the above reasons. In this part we will show that the quality factor depends simply on two starting materials. Because the properties of these two materials are known, it is reasonable to treat them as the multiphase situation. Using the dielectric constants and the quality factors of two starting ceramics, we will show that the quality factor versus volume molar ratio also obeys equation (18). To illustrate better comparison, these 9 examples are grouped by dielectric constant ratio between two ceramics. The *k* value of each group will be obtained through fitting the reported dielectric constant with equation (9). The examples and their corresponding *k* values are set in Group I (*Rε*_*r*21_ ~ 1–2): 1. Ca_2/5_Sm_2/5_TiO_3_-Li_1/2_Nd_1/2_TiO_3_, *k* = 0.95^17^; 2. Ba_2_CaWO_6_-Ba_2_SrWO_6_, *k* = 4^18^; 3. ZnNb_2_O_6_-MgTa_2_O_6_, *k* = 10^19^; 4. Ca_4/5_Sr_1/5_TiO_3_-Li_1/2_Nd_1/2_TiO_3_, *k* = 5^20^; Group II (*Rε*_*r*21_ ~ 2–8): 5. Ca_3/5_La_0.8/3_TiO_3_-CaMg_1/3_Nb_2/3_O_3_ *k* = −1.1^21^; 6. CaTiO_3_- Li_1/2_Sm_1/2_TiO_3_ *k* = 2.5^22^; 7. CaTiO_3_-NdAlO_3_, *k* = −1.35^23^; 8. CaTiO_3_- SrMg_1/3_Nb_2/3_O_3_ *k* = −0.15^24^; 9. BaNd_2_Ti_4_O_12_-NdAlO_3_, *k* = 4^4^. Examples of *Rε*_*r*21_ smaller than 1 can be transferred to be larger than 1 by exchange two materials’ orders. Figure 4(a)–(b) compare the reported experimental dielectric constants of these 9 examples and those calculated by equation (9). In Fig. 4(a), only one dot deviates from the plot with an acceptable deviation less than 10%. In Fig. 4(b), all data distribute around the lines. Figure 4(c)–(d) displays the experimental and equation (18) computed relative quality factor. Compare Fig. 4(b) and (d), the deviation between the reported data and computed results of CaTiO_3_-Sr(Mg_1/3_Nb_2/3_)O_3_ seems larger than other groups. As claimed at the beginning of the work, their samples are probably not well sintered. In addition, the size of samples in their report may also change while this study assumes a constant diameter. As a result, the resonant frequency deviates from the assumption and the relative quality factor differs from the plot. Even though deviations exist, their reported data obey equations (9) and (18). In general, all reported quality factors follow well equation (18), which infers that the quality factor is mainly determined by the properties of two starting materials. Therefore, the solid solution can be treated as the multiphase. The precise quality factor computation as a function of their volume molar ratio is achievable. In the next part, equivalent ionic substitution will also be treated as the multiphase.

### Equivalent ionic substitution examples

**Figure 5:**
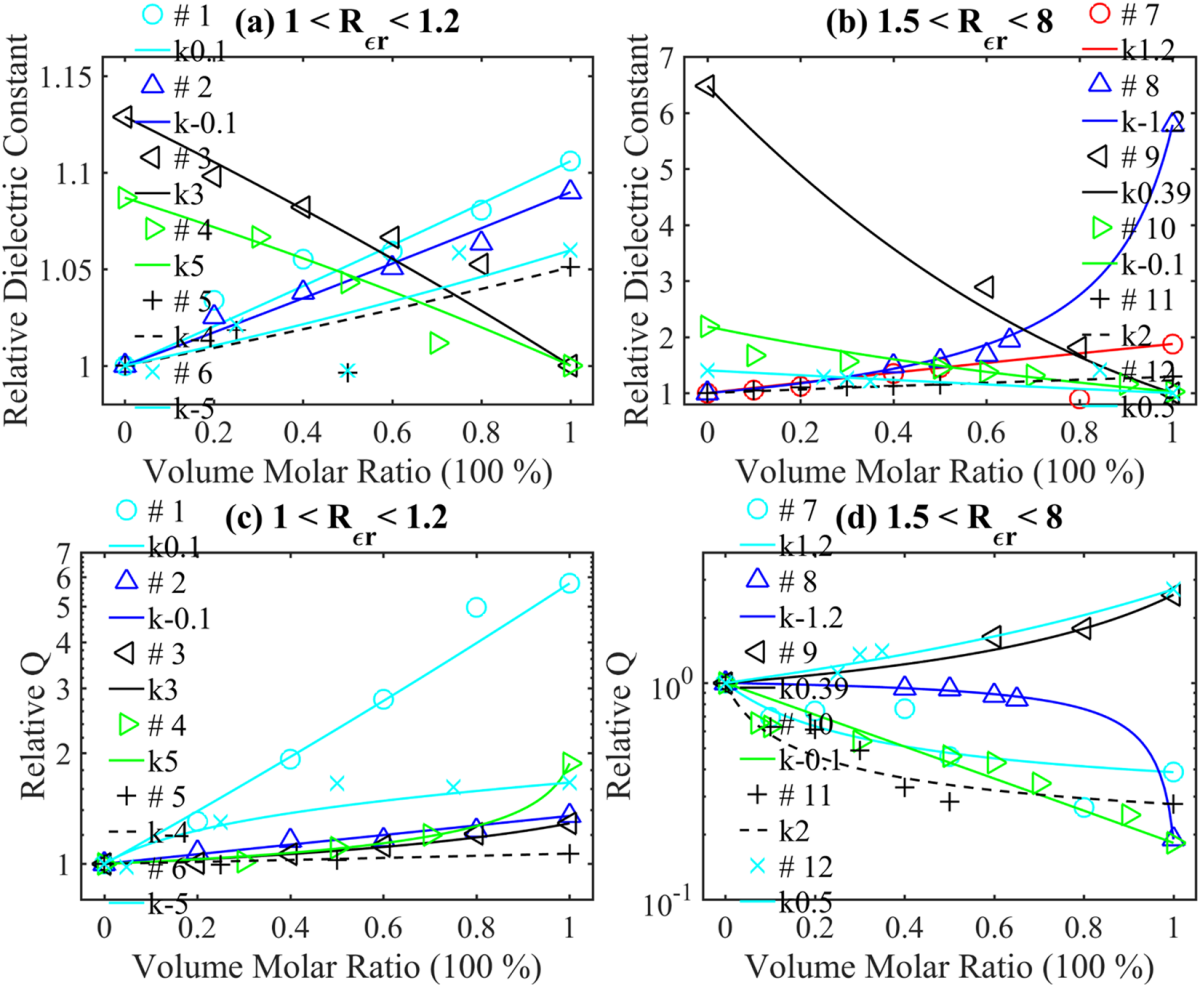
Equivalent ionic substitution examples: (**a**–**b**) the comparison between the reported relative dielectric constants (individual samples) and calculation from Equation (17) (solid lines); (**c**–**d**) the comparison between the reported relative quality factors (individual samples) and calculation from Equation (18) (solid lines). (**a**) and (**c**) # 1. blue-green circles and lines, *k* = 0.1, Ni_1-x_Zn_x_Nb_2_O_6_; 2. blue triangles and lines, *k* = −0.1, Mg_1-x_Zn_x_Al_2_O_4_; 3. black triangles and lines, *k* = 3, Li_2_Co_1-x_Zn_x_Ti_3_O_8_; 4. green triangles and lines, *k* = 5, (Mg_0.95_Ni_0.05_)_4_(Nb_1-x_Ta_x_)_2_O_9_; 5. black + and dashed lines, *k* = −4, Ba_8_Ta_6_(Ni_1-x_Zn_x_)O_24_; 6. × and blue-green lines, *k* = −5, Ba_3.9_(Sm_1-x_Nd_x_)_10.1_Ti_18_O_54_); (**b**) and (**d**) # 7. red circles and lines in (**b**) and blue-green circles and lines in (**d**), *k* = 1.2, Ca_1-x_Sr_x_TiO_3_; 8. blue triangles and lines, *k* = −1.2, CaTi_1-x_(Mg_1/2_Nb_1.2_)_x_o_3_; 9. black triangles and lines, *k* = 0.39, CaTi_1-x_Zr_x_O_3_; 10. green triangles and lines, *k* = −0.1, Ca_1-x_(Li_1/2_Nd_1/2_)_x_TiO_3_; 11. black + and dashed lines, *k* = 2, Mg_1-x_Zn_x_TiO_3_; 12. purple × and lines, *k* = 0.5, Mg(Ta_1-x_Nb_x_)_2_O_6_.

The equivalent ionic substitution can be categorized into two conditions depending on whether the properties of the both materials are known^34–36,44–50^. Sometimes, only properties of one starting material are given^34,36^. For example, with the Bi^3+^ substitution with Nd^3+^ in Ba_6-3x_Nd_8+2x_Ti_18_O_54_, it exhibits properties change compared to Ba_6-3x_Nd_8+2x_Ti_18_O_54_^34^. In this situation, only properties of Ba_6-3x_Nd_8+2x_Ti_18_O_54_ are known^1,34^. In other situations, the properties of the two materials are known^44–50,58^. For example, in Li_2_Co_1-x_Zn_x_Ti_3_O_8_ ceramics, the properties of both Li_2_ZnTi_3_O_8_ and Li_2_CoTi_3_O_8_ are known^45^. As the substitution molar ratio increases, the microstructure or the parameters of the original molecular will systemically change. Therefore, the quality factor versus the substitution content is currently attributed to interfacial mismatch^44^, mode vibration^35,58^, phase constitution^45,50^, relative density^46^, grain size^47^, dielectric constant^48^, and crystal structure^59^ *etc*. The interfacial mismatch can be related to grain size which corresponds to the two-dimensional defect leading to the dielectric loss^44^. The vibration modes can be determined via the Rama measurement^35,58^. However, this usually sweeps a wide range of frequencies, and once the resonator is accomplished the resonant frequency of the microwave dielectric ceramics is determined. As a result, it is illogical to estimate the dielectric loss in other frequencies. In addition, the low relative density or high porosity is neglectable for well sintered ceramics. Through equations (8) and (19), *Q* × *f* is obviously independent of the dielectric constant, while the quality factor *Q* depends on it. In this paper, we will discuss the situation where the properties of both initial materials are known. We will show that the quality factor variation obeys equation (18). To exhibit better comparison, 12 examples are grouped by dielectric constant ratio of the two initial ceramics. The *k* value of each group will be obtained through fitting the reported dielectric constant with equation (9). The examples and their corresponding *k* values are grouped in Group I (*Rε*_*r*21_ ~ 1–1.2): 1. Ni_1-x_Zn_x_Nb_2_O_6_, *k* = 0.1^44^; 2. Mg_1-x_Zn_x_Al_2_O_4_, *k* = −0.1^35^; 3. Li_2_Co_1-x_Zn_x_Ti_3_O_8_, *k* = 3^45^; 4. (Mg_0.95_Ni_0.05_)_4_(Nb_1-x_Ta_x_)_2_O_9_, *k* = 5^46^; 5. Ba_8_Ta_6_(Ni_1-x_Zn_x_)O_24_ *k* = −4^47^; 6. Ba_3.9_(Sm_1-x_Nd_x_)_10.1_Ti_18_O_54_ *k* = −5^48^; Group II (*Rε*_*r*21_ ~ 1.3–7): 7. Ca_1-x_Sr_x_TiO_3_, *k* = 1.2^49^; 8. CaTi_1-x_(Mg_1/2_Nb_1.2_)_x_o_3_, *k* = −1.2^50^; 9. CaTi_1-x_Zr_x_O_3_, *k* = 0.39^58^; 10. Ca_1-x_(Li_1/2_Nd_1/2_)_x_TiO_3_, *k* = −0.1^59^; 11. Mg_1-x_Zn_x_TiO_3_, *k* = 2^53,61^; 12. Mg(Ta_1-x_Nb_x_)_2_O_6_, *k* = 0.5^31^. Examples of *Rε*_*r*21_ smaller than 1 can be transferred to larger than 1 by exchange the two initial materials’ orders. Figure 5(a)–(b) show the comparison between the reported experimental dielectric constant and the plot of prediction of equation (9) of these 12 groups. In Fig. 5(a), the y-axe is in the range of 1~1.14, and some points seem deviate from the plot. However, the largest relative deviation is as low as 4%. In Fig. 5(b), all reported experimental data fit to the plot of the prediction. It is conclusive that all reported experimental results follow well the prediction of equation (9), which confirms the effectiveness of equation (9). Figure 5(c)–(d) compare the reported experimental relative quality factor to the calculated result via equation (18). All the reported quality factors match well with the results computed via equation (18) except for the data of Ca_1-x_Sr_x_TiO_3_ with a volume molar ratio of 0.8. Comparing its dielectric constant at this point in Fig. 5(b), the deviation is due to its dielectric constant disagreement. The agreement between the previous experimental data and the prediction of equation (18) indicates that the quality factor of equivalent ionic substitution is also determined by their dielectric constant and dielectric constant index *k*. Therefore, using the dielectric constants and the quality factors of the two starting materials, one can precisely compute the quality factor of the equivalent ionic substituted materials as a function of the volume molar ratio.

### Inequivalent ionic substitution

By the Maxwell Equations, the dielectric loss actually consists of both the part described in equation (7) and the materials conductivity. Considering both parts, the dielectric loss of a material is^39,62^

20 $$\tan \,{\delta }=\frac{\varepsilon ^{\prime\prime} }{\varepsilon ^{\prime} }+\frac{\sigma }{\omega \varepsilon ^{\prime} }$$

The examples discussed in the above actually ignored the impacts from the material conductivity, $\sigma$. In some situations, the conductivity originates from the inequivalent substitution is so high that one has to turn to equation (20) and ignore the first part on the right side.

## Conclusions

In conclusion, based on a revised dielectric constant equation, we proposed a new formula that could precisely describe and predict the quality factor of multiphase, solid solution, and equivalent ionic substituted ceramics. The prediction of this formula agrees very well with the previously reported experimental data. We found that the quality factor of mixed ceramics is determined by the quality factor, dielectric constants, and dielectric constant index *k* of each initial component.
